# The Effects of Facial Attractiveness and Familiarity on Facial Expression Recognition

**DOI:** 10.3389/fpsyg.2019.02496

**Published:** 2019-11-25

**Authors:** Jinhui Li, Dexian He, Lingdan Zhou, Xueru Zhao, Tingting Zhao, Wei Zhang, Xianyou He

**Affiliations:** ^1^School of Psychology, South China Normal University, Guangzhou, China; ^2^Academy of Educational Science Talent Capital Base, Beijing Institute of Education, Beijing, China; ^3^School of Health Management, Guangzhou Medical University, Guangzhou, China; ^4^Center for Studies of Psychological Application, South China Normal University, Guangzhou, China; ^5^Guangdong Key Laboratory of Mental Health and Cognitive Science, South China Normal University, Guangzhou, China

**Keywords:** dynamic theory of face perception, face, attractiveness, expression recognition, familiarity

## Abstract

The classic theory of face perception holds that the invariant (e.g., identity and race) and variant (e.g., expression) dimensions of face information are independent of one another. Two separate neural systems are involved in face processing. However, the dynamic theory of face perception indicates that these two neural systems interact bidirectionally. Accordingly, by using the emotion categorization task and morph movies task, we investigated the influence of facial attractiveness on facial expression recognition and provided further evidence supporting the dynamic theory of face perception in both the static and dynamic contexts. In addition, this research used familiar celebrities (including actors, television personalities, politicians, and comedians) and explored the role of familiarity in face perception. In two experiments, the participants were asked to assess the expressions of faces with different levels of attractiveness and different levels of familiarity. We found that regardless of being in a static or dynamic face situation, happy expressions on attractive faces can be recognized more quickly, highlighting the advantage of happy expression recognition. Moreover, in static and dynamic familiar face situations, familiarity has a greater impact on expression recognition, and the influence of attraction on expression recognition may be weakened or even unaffected. Our results show that facial attractiveness influences the recognition of facial expressions in both static and dynamic contexts and highlight the importance of familiarity in face perception.

## Introduction

Facial expressions can convey information regarding individuals’ emotions and social intentions, which is of great importance for social interaction. The rapid and correct identification of facial expressions is a necessity for successful social interaction. A classic cognitive model of face perception emphasizes the difference between the processes involved in the recognition of identity and those involved in the identification of expression (Bruce and Young, 1986). Based on this model, Haxby et al. (2000) proposed a model for the workings of this system that emphasized a difference between the indication of constant and variant sides of faces. The representation of the constant characteristics of faces (e.g., sex, race, and identity) underlies the recognition of individuals, whereas the representation of the variant characteristics of faces (e.g., expression) underlies the perception of information that promotes social interaction.

In recent years, classic models of face perception have increasingly been challenged (Calder and Young, 2005; Hugenberg and Sczesny, 2006; Becker et al., 2007; Fisher et al., 2016). For example, one study found that the processing of facial identity and expression involves functional interactions and that their independence is not absolute (Calder and Young, 2005). This study proposed that the invariant and variable features of faces may be encoded by the same perceptual characterization system, followed by separation. Hugenberg and Sczesny (2006) found that participants could identify angry facial expressions faster in male faces than female faces. Becker et al. (2007) suggested that decisions regarding the gender of a face and facial expressions are not separate and found that subjects were faster and more accurate in discovering angry expressions on male faces and happy expressions on female faces. Fisher et al. (2016) identified the interaction between facial identity and expression. Similarly, other studies have concluded that there are different degrees of overlap between the brain regions that process face information (e.g., Ganel et al., 2005; Fox et al., 2009; Redfern and Benton, 2017). Specifically, Fox et al. (2009) found that the processes involved in facial identity and expression are not completely independent and that different degrees of overlap exist between the brain regions processing face information. Ganel et al. (2005) identified an interactive network responsible for the processing of expression and identity. Redfern and Benton (2017) used an identification task and concluded that expressions constitute a part of facial identity representation.

Given the debate regarding the classic theory of face perception, Quinn and Macrae (2011) proposed a dynamic theory of face perception. These authors proposed the existence of integrated processing pathways responsible for face processing. Facial characteristics (including invariant and variant characteristics) are processed in a multidimensional face coding system. The facial structure is coded in the primary stage; then, more sophisticated information is processed in the same dynamic system, and there is a general interaction. This view that facial characteristics are processed in a multidimensional face coding system has been confirmed by many studies (Ganel et al., 2005; Freeman et al., 2008; Fox et al., 2009). For example, Fox et al. (2009) found that facial identity and expression are not processed completely independently and that there are different degrees of overlap between the brain regions involved in face information processing. Other evidence suggesting that the invariant dimension of face information affects participants’ recognition of face’s variant dimensions has been reported (Craig et al., 2012; Fitousi and Wenger, 2013; Smith et al., 2017; Craig and Lipp, 2018).

Although previous research has typically focused on how invariant and variant face-related information is incorporated into judgments of facial expression, limited research has considered the relevance and importance of facial attractiveness. Face processing theories have paid minimal attention to the role of attractiveness and how attractiveness relates to other facial attributes. In the field of face perception, researchers have incorporated facial attractiveness into the invariant dimension of face information (Rhodes, 2006; Winston et al., 2007; Iaria et al., 2008). For example, Iaria et al. (2008) found that the fusiform gyrus (FFA) is activated when making facial attractiveness judgments and that the FFA mainly processes the invariant dimensions of faces. Additionally, noted attractiveness is based more on the temporally invariant aspects than the dynamic aspects of facial structure. Rhodes (2006) suggested that facial attractiveness may be more similar to the properties of identity and gender in terms of its processing demands. The attractiveness of a face is a salient social signal that reflects the overall effect of all physical attributes of a face.

Several studies have concluded that our perception of the attractiveness of a face is moderated by its facial expression (Magda and Goodwin, 2008; Tracy and Beall, 2011; Golle et al., 2014; Sutherland et al., 2017). In these studies, the participants perceived faces as more attractive when the facial expression was happy as opposed to other expressions. The apparent link between attractiveness and facial expression has been strengthened by recent neurological evidence emphasizing increased activity in the medial orbitofrontal cortex (OFC) during the presentation of stimuli that are attractive and positively valenced (O’Doherty et al., 2003). Sun et al. (2015) used the event-related potential (ERP) method to explore whether facial attractiveness and facial expression are processed similarly in the brain. They found that facial attractiveness and facial expression were separately embodied by two early components, i.e., N170 and P2, while their interaction effect was embodied by the late positive potential (LPP), which is a late component (Sun et al., 2015). Given that attractiveness is affected by facial expression recognition and that there is an overlapping brain region involved in facial attractiveness and facial expression recognition, we propose that attractiveness also affects expression recognition.

To the best of our knowledge, few studies have explored whether facial attractiveness contributes to facial expression, and the results of these studies are not consistent. Taylor and Bryant (2016) found that there was no interaction between facial attractiveness and expression. In their study, the authors asked the participants to categorize different facial expressions (happy, neutral, or angry) that varied with respect to facial attractiveness (attractive or unattractive). Their results suggested that facial attractiveness does not play a significant role in the judgment of happy or angry facial expressions. An earlier study also found no interaction between facial attractiveness and facial expression in the ratings of emotion valence (Jaensch et al., 2014). In contrast, Lindeberg et al. (2018) used an emotion category task and found that facial social classification cues influenced emotion perception. Thus, the authors found an interaction between facial attractiveness and expression. Specifically, they identified a greater happy face advantage resulting in more positively evaluated attractive faces than unattractive faces. Golle et al. (2014) indicated that the attractiveness of a face could affect the assessment of the happy expression. We suspect that these different experimental results may be caused by different experimental paradigms selected for different experiments. Taylor and Bryant (2016) and Lindeberg et al. (2018) used an emotional classification task in an experiment, but Lindeberg et al. (2018) used a larger sample size. Golle et al. (2014) utilized two alternative forced choice (2AFC) paradigms. It is also possible that different experiments use angry expressions as negative expressions and that angry expressions are often confused with other expressions (Taylor and Jose, 2014), leading to inconsistent conclusions in different studies. Although Lindeberg et al. (2018) verified that face attractiveness affects expression recognition, the findings of their study are inconsistent with the findings reported by Taylor and Bryant (2016). Therefore, more evidence concerning whether facial attractiveness affects facial expression identification should be collected. In addition, the facial expressions used in this research are happy and sad, which are not exactly the same as the happy and angry expressions used by Lindeberg et al. (2018). We used an experiment consistent with Lindeberg et al. (2018) in Experiment 1a. On the one hand, the paradigm investigates whether the recognition of facial expressions is affected by attractiveness. On the other hand, this study is an extension of existing research. The sad expression represents experimental material that expands the range of expressions affected by attractiveness and further verifies the relationship between facial attractiveness and expression recognition.

In addition, previous research illustrates that familiar stimuli prompt diverse positive reactions (Zajonc, 1968; Bornstein, 1989). Many studies have found that familiarity affects the processing of face perception (i.e., facial attractiveness and facial expressions) (Moreland and Beach, 1992; Dubois et al., 1999; Claypool et al., 2007; Carr et al., 2017; Yan et al., 2017). Moreover, studies have shown that there are strong interactions between familiarity and expression recognition (Claypool et al., 2007; Carr et al., 2017). For example, Carr et al. (2017) concluded that familiar faces appear happier and less angry than unfamiliar faces, indicating that familiarity affects facial expression recognition. Claypool et al. (2007) also found the same result. Furthermore, previous studies have examined how multiple social category cues, namely, sex and race (Smith et al., 2017; Craig and Lipp, 2018) and sex and age (Craig and Lipp, 2018), simultaneously moderate expression recognition and provided evidence of the combined influence of these social cues on expression recognition. However, no studies have investigated how facial attractiveness and familiarity simultaneously moderate expression recognition. Thus, in the present research, we manipulate facial familiarity.

More importantly, most existing research concerning facial expression recognition has used static face images (Claypool et al., 2007; Dobel et al., 2008; Carr et al., 2017), whereas in real life, faces are typically seen in motion. In addition, the dynamic context is more ecologically valid. That is, in interpersonal contexts, people’s facial expressions are usually in a dynamic situation (Niedenthal et al., 2000; Rubenstein, 2005; Ishii et al., 2011). Therefore, in this research, we presented both static and dynamic faces to subjects to judge facial expressions.

As mentioned above, the present research uses the emotion categorization task (see Bijlstra et al., 2010; Taylor and Bryant, 2016; Lindeberg et al., 2018) and morph movies task (see Niedenthal et al., 2000; Hugenberg and Bodenhausen, 2003; Bijlstra et al., 2014) in static and dynamic contexts. Accordingly, we investigate the extent to which attractiveness and familiarity influence facial expression processing. We conduct two experiments to explore this problem. According to the dynamic theory of face perception, if the attractiveness associated with face information can affect the processing of expression recognition, the processing of facial attractiveness and expression recognition are dependent on one another. However, according to the classic theory of face perception, if the facial attractiveness related to face information does not affect the processing of expression recognition, the processing of facial attractiveness and expression recognition are independent of one another. Based on behavioral evidence suggesting that attractive faces are often associated with positive personality characteristics (Dion et al., 1972; Golle et al., 2014; Wang et al., 2015; Lindeberg et al., 2018), we hypothesize that participants can recognize the happy expressions of attractive faces more quickly and that the advantages of happy expression recognition do not apply to unattractive faces in either a static context or a dynamic context (Experiments 1a and 1b). In addition, in accordance with previous studies (Carr et al., 2017; Smith et al., 2017; Craig and Lipp, 2018; Lindeberg et al., 2018), we anticipate that if familiarity has a greater impact on facial expression recognition, under the familiar face condition, the impact of attractiveness on facial expression recognition may be weakened or even unaffected. Similarly, compared to the other conditions, if familiarity and attractiveness together affect expression recognition, happy expressions on familiar attractive faces can be identified more quickly.

In general, the main aim of this research is to investigate whether facial attractiveness affects expression recognition in both static (Experiment 1a) and dynamic (Experiment 1b) contexts. This research also explores how familiarity and facial attractiveness can affect expression recognition in static (Experiment 2a) and dynamic (Experiment 2b) contexts.

## Experiment 1a

In Experiment 1a, we sought to determine whether facial attractiveness influences facial expression recognition in static faces. We predicted an interaction between facial attractiveness and expression recognition, i.e., we predicted that the participants would recognize the happy expressions on attractive faces more quickly and that the advantages of happy expression recognition would not apply to unattractive faces.

### Method

#### Participants

According to selection criteria for participants used in previous research (Hugenberg, 2005; Taylor and Bryant, 2016), we recruited a total of 30 Chinese university students from South China Normal University (21 females, *M* = 21.00 years, *SD* = 1.39 years) to participate in an emotion categorization task. Based on a *post hoc* power analysis (*α* of 0.05, *η*^2^ = 0.50, G*Power 3.1), we found that this sample size yielded a high power of 1 − *β* = 0.85. The participants classified happy and sad emotional expressions displayed on both attractive and unattractive faces. All participants were right-handed and had normal or corrected-to-normal vision. The participants were paid for their participation. New participants were recruited for each experiment. Once the participants had completed both experimental blocks, they were thanked and debriefed.

#### Ethics Statement

This research was implemented following approval by the Institutional Review Board of South China Normal University, and written informed consent was obtained from all participants in accordance with the Declaration of Helsinki.

#### Materials and Procedure

##### Materials

We collected 100 photographs of unfamiliar Chinese male and female faces (50 male) with a neutral emotional expression from the Baidu website,^1^ which is a public website. The search keyword was images (Chinese photo). These materials are intended to be used for free in research and are not to be used for commercial purposes. The pictures of real human faces have often been utilized in previous studies concerning face perception. Such pictures have high ecological validity (Hugenberg, 2005; Sun et al., 2015), although controlling for their confounding elements (e.g., skin texture and hair color) is challenging. Here, we employed fabricated facial stimuli to control for variables of no interest (Bijlstra et al., 2014; Sun et al., 2015). The images were edited by using FaceGen software^2^ to obtain a virtual 3D picture of each real face. The software permitted the manipulation of the expression without changing the facial physiognomies of the targets. Fifteen additional Chinese participants (seven females) were asked to rate the level of attractiveness and familiarity of each FaceGen version of a face on a 7-point scale. Twenty facial images (10 males) with varying levels of attractiveness were selected as the experimental stimuli. The ratings of the attractive faces (*M* = 4.31, *SD* = 0.45) differed from the ratings of the unattractive faces (*M* = 2.47, *SD* = 0.64, *F*(1, 18) = 55.10, *p* < 0.01); however, there was no difference in the ratings of familiarity between the attractive faces (*M* = 3.05, *SD* = 0.25) and unattractive faces (*M* = 2.87, *SD* = 0.25, *F*(1, 18) = 2.63, *p* = 0.12). Each of the 20 stimulus faces was then further manipulated to create the following two versions: a version with a distinctly happy expression and a version with a distinctly sad expression. All images were sized at 400 × 400 pixels and presented on a black background. We also balanced the factors that might affect the participants’ responses, such as the greyscale and color. By using a series of paired *t*-tests, we found that there was no significant difference in the levels of attractiveness and familiarity between the neutral and happy faces in the same stimuli face, *t*(19) = 1.28, *p* = 0.22 and *t*(19) = 1.88, *p* = 0.08, respectively. There was no significant difference in the levels of attractiveness and familiarity between the neutral and sad faces in the same face stimuli, *t*(19) = 0.43, *p* = 0.67 and *t*(19) = 1.97, *p* = 0.06, respectively. There was no significant difference in the levels of attractiveness and familiarity between the happy and sad faces in the same face stimuli, *t*(19) =1.49, *p* = 0.15 and *t*(19) =0.15, *p* = 0.88, respectively.

##### Procedure

**Figure 1:**
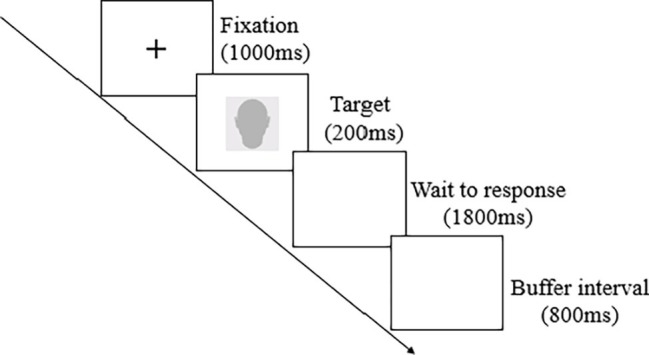
Procedure of the emotion categorization task. The images presented on the screen were all virtual 3D pictures of real faces used in the experiments, but in this figure, we used blank profile pictures instead of a real facial stimulus due to privacy concerns.

The emotion categorization task was consistent with existing research (Bijlstra et al., 2010) (see Figure 1). In these tasks, the stimuli were presented by using E-prime 1.1 software. The participants were seated at desks with Lenovo PCs approximately 60 cm from the display computer in a quiet room. The stimuli were presented on a 23.8-inch LEN monitor with a screen resolution of 1,024 × 768 pixels. Each trial consisted of a fixation cross, which was presented for 1,000 ms, followed by a face that exhibited one emotional expression for 200 ms. The participants were asked to identify the expression displayed in the images of the attractive and unattractive faces by pressing the “F” key for happy or the “J” key for sad; the reaction screen disappeared automatically after 1,800 ms, and there was one picture per trial. The task consisted of three blocks, namely, one practice block and two experimental blocks. The pictures used in the practice block were not used in the formal experimental blocks. Each experimental block consisted of all 20 photos (only one version of each face) exhibited once in random order. The duration of the experiment per participant was approximately 8 min.

### Results and Discussion

Before the analysis, four participants with more than 25% missing data (32.5, 27.5, 35, and 32.5% of the trials) were excluded from further analysis (Lindeberg et al., 2018). Therefore, the final analysis included data from 26 participants. Meanwhile, errors (incorrect button presses; 5.29% of the trials), invalid responses (0.29% of the trials), and outliers (response times that deviated from an individual’s mean by more than 3 *SD*; 1.06% of the trials) were excluded from the response time analysis. SPSS 22.0 software was used for the data analysis, and the analysis only included correct trials.

**Figure 2:**
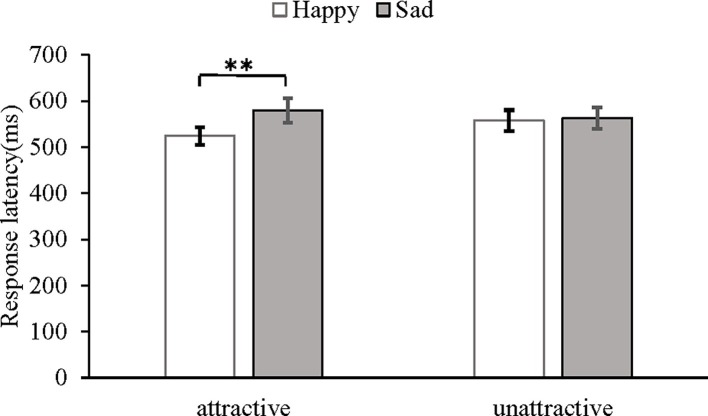
Mean response latency and SEs (standard errors) in milliseconds under different conditions of attractiveness and expression as measured by the emotion categorization task. **p < 0.01.

The primary dependent variable in this study was the mean response time required to categorize the emotional expressions. Due to the skewed distribution of the response latencies (Whelan, 2008; Bijlstra et al., 2010; Lo and Andrews, 2015), all analyses were performed based on the log-transformed response latencies (Bijlstra et al., 2010, 2014; Lo and Andrews, 2015). To facilitate the interpretation of our findings, we report the mean response latencies in untransformed milliseconds. The mean log-transformed response latencies were subjected to a 2 (attractiveness: attractive vs. unattractive) × 2 (expression: happy vs. sad) mixed-model ANOVA. In the statistical test results, the spherical test was *p* = 0.15 > 0.05, indicating that the data satisfy the spherical hypothesis. This analysis revealed a main effect of expression, *F*(1, 25) = 7.36, *p* < 0.05, *η*^2^ = 0.23. The main effect of attractiveness was not significant (*F =* 0.81, *p =* 0.38). More importantly, the interaction between attractiveness and expression was significant, *F*(1, 25) =7.05, *p* < 0.01, *η*^2^ = 0.22 (see Figure 2), and a follow-up paired *t*-test confirmed that the happy expression (*M* = 524, *SD* = 97) was recognized faster than the sad expression (*M* = 579, *SD* = 136) on the attractive faces, *t*(25) = 3.53, *p* < 0.01, 95% confidence interval (CI) [0.02, 0.06]. No difference was found in the response latencies between the happy (*M* = 557, *SD* = 119) and sad (*M* = 563, *SD* = 118) expressions displayed on the unattractive faces [*t*(25) = 0.62, *p =* 0.54, 95% CI (−0.01, 0.03)].

#### Accuracy

We analyzed the error rate by the same method used to analyze the reaction time and found that the main effect of attractiveness was significant, *F*(1, 25) = 30.24, *p* < 0.01, *η*^2^ = 0.55; the main effect of expression was significant, *F*(1, 25) = 5.20, *p* < 0.05, *η*^2^ = 0.17; and the interaction between attractiveness and expression was significant, *F*(1, 25) =143.87, *p* < 0.001, *η*^2^ = 0.85. In the follow-up paired sample *t*-test, the error rate of the happy expression recognition on the attractive faces, *t*(25) =11.92, *p* < 0.001, and sad expression recognition on the unattractive faces, *t*(25) = 4.24, *p* < 0.001, was lower.

The results of Experiment 1a show an interaction between facial attractiveness and expression recognition, suggesting that facial attractiveness has an effect on expression recognition in static emotional face paradigms. Specifically, the participants were able to recognize the happy expressions on the attractive faces more quickly, and under the unattractive face condition, there was no difference between happy expression recognition and sad expression recognition. This finding shows that attractiveness affects expression recognition. Moreover, the results correspond with the dynamic theory of face perception.

## Experiment 1b

The purpose of Experiment 1b was to explore whether the influence of facial attractiveness on expression recognition found in Experiment 1a appeared in a dynamic context. Simultaneously, to enhance the ecological validity of the experimental situation, this experiment used dynamic faces to replicate and extend the findings of Experiment 1a. Similarly, we predicted that the participants could recognize happy expressions on the attractive faces more quickly and that the advantages of happy expression recognition do not apply to unattractive faces.

### Method

#### Participants and Design

In total, 33 new Chinese university students from South China Normal University (23 females, *M* = 21.12 years, *SD* = 1.63 years) completed an emotion morph movies task in which the participants watched short film clips of attractive and unattractive faces that changed from neutral-to-happy or sad expression. Based on a *post hoc* power analysis (*α* of 0.05, *η*^2^ = 0.50, G*Power 3.1), we found that this sample size yielded a high power of 1 − *β* = 0.88. All participants were right-handed and had normal or corrected-to-normal vision.

#### Materials and Procedure

##### Materials

We used the FaceGen versions of the face models from Experiment 1a to create short film clips by FataMorph.^3^ We created two film clips (neutral-to-happy and neutral-to-sad) for each of the 20 models. Furthermore, four film clips were established and used to familiarize the participants with the experimental task in an initial practice block.

##### Procedure

The participants were seated in individual cubicles and informed that they would be presented with short film clips of faces that demonstrate a neutral expression that changed into a second expression. The morph movies task was consistent with existing studies (Niedenthal et al., 2000; Bijlstra et al., 2014). We instructed the participants to watch each film clip of neutral expressions and press “F” or “J” the moment that they detected the onset of a happy or sad expression in a face. Each clip was shown once per test block; the presentation order of the film clips was randomized for each participant. The duration of the experiment per participant was approximately 15 min.

### Results and Discussion

Before the analysis, one participant was excluded due to missing data exceeding 25% (30% of the trials). Meanwhile, errors (incorrect button presses; 5.31% of the trials) and invalid responses (1.95% of the trials) were excluded from the response time analysis.

**Figure 3:**
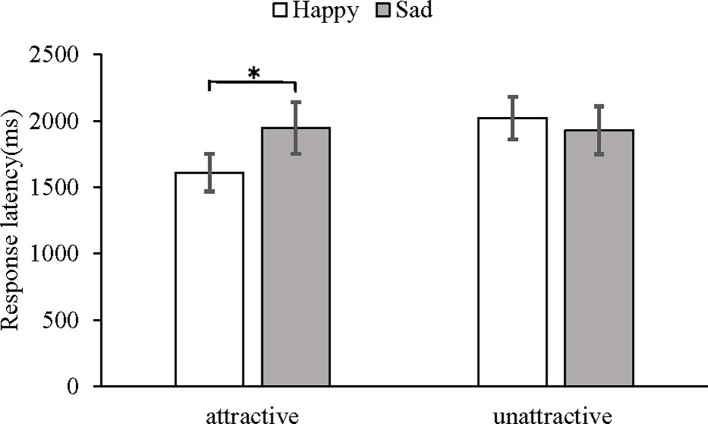
Mean response latency and SEs in milliseconds under different conditions of attractiveness and expression as measured by the morph movies task. *p < 0.05.

The mean log-transformed response latencies were subjected to a 2 (attractiveness: attractive vs. unattractive) × 2 (expression: happy vs. sad) mixed-model ANOVA. The spherical test *p* = 0.59 > 0.05 in the statistical test results indicated that the data satisfied the spherical hypothesis. This analysis showed a main effect of attractiveness, *F*(1, 31) = 6.19, *p* < 0.05, *η*^2^ = 0.17. The main effect of expression was not significant, *F*(1, 31) = 0.63, *p* = 0.43. More importantly, the interaction between attractiveness and expression was significant, *F*(1, 31) = 7.78, *p* < 0.05, *η*^2^ = 0.20 (see Figure 3), and a follow-up paired *t*-test confirmed that recognition of a happy expression (*M* = 1,611, *SD* = 805) was faster than of a sad expression (*M* = 1,948, *SD* = 1,108) on the attractive faces, *t*(31) = 2.37, *p <* 0.05, 95% CI [0.01, 0.13]. No difference in the response latencies was found between the happy (*M* = 2,022, *SD* = 911) and sad (*M* = 1,930, *SD* = 1,018) expressions displayed on the unattractive faces [*t*(31) = 1.52, *p =* 0.14, 95% CI (−0.01, 0.09)].

#### Accuracy

An analysis of the error rate revealed that the main effect of facial attractiveness was significant, *F*(1, 31) = 5.92, *p* < 0.05, *η*^2^ = 0.16; the main effect of expression was not significant, *F*(1, 31) = 0.55, *p* = 0.46; and the interaction between attractiveness and expression was significant, *F*(1, 31) = 79.76, *p* < 0.001, *η*^2^ = 0.72. In the follow-up paired sample *t*-test, the happy expression recognition on the attractive faces, *t*(31) = 5.83, *p* < 0.001, and the error rate of sad expression recognition on the unattractive faces, *t*(25) = 6.26, *p* < 0.001, were lower.

The results of Experiment 1b revealed an interaction between facial attractiveness and expression; this finding suggests that facial attractiveness has an effect on expression recognition in dynamic emotional face paradigms.

Experiments 1a and 1b provide confirmatory evidence that the ability to process facial expressions is influenced by facial attractiveness. Studies have shown that familiarity affects the information processing of faces (Zajonc, 1968; Claypool et al., 2007; Carr et al., 2017). Previous studies have examined how multiple social category cues, sex and race (Smith et al., 2017; Craig and Lipp, 2018), and sex and age (Craig and Lipp, 2018) simultaneously moderate expression recognition and provided evidence of the combined influence of these social cues on expression recognition. However, no study has examined how facial attractiveness and familiarity simultaneously moderate expression recognition. Therefore, Experiments 2a and 2b were designed to explore whether facial attractiveness and familiarity simultaneously moderate expression recognition in static and dynamic contexts, respectively.

## Experiment 2a

We aim to explore whether facial attractiveness and familiarity simultaneously moderate expression recognition in a static face display. We predict that if the impact of familiarity is greater, the role of attractiveness may be diminished under the familiar face condition. If familiarity and attractiveness combine to affect expression recognition, familiar and attractive happy expressions should be identified more quickly.

### Method

#### Participants

Previous studies have observed the reliable effects of facial attributes, such as sex and race, on the happy face advantage in samples of approximately 30 participants (e.g., Craig and Lipp, 2018). Thus, in total, 32 new Chinese university students from South China Normal University (26 females, *M* = 21.19 years, *SD* = 1.91 years) completed an emotion categorization task consistent with Experiment 1a. Based on a *post hoc* power analysis (*α* of 0.05, *η*^2^ = 0.50, G*Power 3.1), we found that this sample size yielded a high power of 1 − *β* = 0.87. All participants were right-handed and had normal or corrected-to-normal vision.

#### Materials and Procedure

##### Materials

We used the 20 FaceGen versions of facial images from Experiment 1a as unfamiliar faces. In addition, we used 20 faces of familiar celebrities (10 males and 10 females) with varying levels of attractiveness from a range of prominent figures in China, including actors, television personalities, politicians, and comedians. The creation of the two versions of expression on these faces was consistent with the method described in Experiment 1a. Nineteen additional Chinese participants (10 females) were asked to rate the level of attractiveness and familiarity of each FaceGen version of each face. Each of the 40 stimulus faces was then further manipulated to produce the following two versions: one version with a distinctly happy expression and a second version with a distinctly sad expression. Using a series of paired *t*-tests, we found that there were no significant differences in the levels of attractiveness or familiarity between the neutral and happy faces in the same face stimuli, *t*(39) = 1.63, *p* = 0.11 and *t*(39) = 1.18, *p* = 0.24, respectively. There were no significant differences in levels of attractiveness or familiarity between the neutral and sad faces of the same face stimuli, *t*(39) = 1.53, *p* = 0.14 and *t*(39) = 0.84, *p* = 0.41. Moreover, there were no significant differences in the levels of attractiveness or familiarity between the neutral and sad faces in the same face stimuli, *t*(39) = 1.53, *p* = 0.14 and *t*(39) = 0.84, *p* = 0.41, respectively. In addition, there were no significant differences in the levels of attractiveness or familiarity between the happy and sad faces in the same face stimuli, *t*(39) = 0.03, *p* = 0.98 and *t*(39) = 0.03, *p* = 0.98, respectively.

Overall, in the attractive group, the difference in the level of attractiveness between the familiar faces (*M* = 4.50, *SD* = 0.33) and unfamiliar faces (*M* = 4.31, *SD* = 0.45) in the neutral state was not obvious, *F*(1, 18) = 1.17, *p* = 0.29, and the difference in familiarity (familiar faces, *M* = 4.42, *SD* = 0.40; unfamiliar faces, *M* = 3.05, *SD* = 0.25) was significant, *F*(1, 18) = 85.08, *p* < 0.001. In the unattractive group, the difference in the level of attractiveness between the familiar faces (*M* = 2.47, *SD* = 0.64) and unfamiliar faces (*M* = 2.37, *SD* = 0.72) in the neutral state was not obvious, *F*(1, 18) =0.10, *p* = 0.77, and the difference in familiarity (familiar faces, *M* = 4.75, *SD* = 0.67; unfamiliar faces, *M* = 2.87, *SD* = 0.25) was significant, *F*(1, 18) = 69.76, *p* < 0.001. In addition, the level of attractiveness in the attractive (*M* = 4.40, *SD* = 0.40) and unattractive (*M* = 2.42, *SD* = 0.66) groups in the neutral state significantly differed, *F*(1, 18) =131.89, *p* < 0.001.

##### Procedure

Experiment 2a closely followed the procedure used in Experiment 1a, except for the face stimuli used.

### Results and Discussion

Before the analysis, seven participants with missing data were excluded from further analysis due to missing more than 25% of the data (32.50, 35.00, 36.25, 36.25, 41.25, 35, and 36.25% of the trials). Therefore, the final analysis included data from 25 participants. Meanwhile, errors (incorrect button presses; 4.25% of the trials), invalid responses (0.05% of the trials) and outliers (response times that deviated from an individual’s mean by more than 3 *SD*; 1.60% of the trials) were excluded from the response time analysis. The primary dependent variable in the present study was the average reaction time to categorize emotional displays on attractive and unattractive faces. The mean log-transformed response latencies were subjected to a 2 × 2 × 2 mixed-model ANOVA devised with the factors of attractiveness (attractive vs. unattractive), expression (happy vs. sad), and familiarity (familiar vs. unfamiliar).

The spherical test *p* = 0.90 > 0.05 in the statistical test results indicated that the data satisfied the spherical hypothesis. The analysis showed no significant main effect of attractiveness [*F*(1, 24) = 0.45, *p =* 0.51] and no significant main effect of expression [*F*(1, 24) = 0.04, *p =* 0.84]. Furthermore, there was no significant main effect of familiarity [*F*(1, 24) = 0.46, *p =* 0.51].

**Figure 4:**
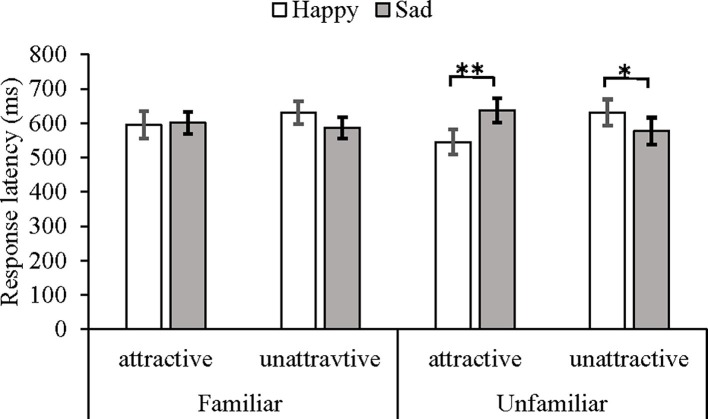
Mean response latency and SEs in milliseconds under different conditions of familiarity, attractiveness, and expression as measured by the emotion categorization task. *p < 0.05, **p < 0.01.

Critically, however, there was a significant three-way interaction among attractiveness, expression, and familiarity, *F*(1, 24) = 4.98, *p* < 0.05, *η*^2^ = 0.17. Under the unfamiliar face condition, the *post hoc* tests with a series of paired *t*-tests showed that the recognition of a happy expression (*M* = 545, *SD* = 180) was faster than that of a sad expression (*M* = 637, *SD* = 182) on the attractive faces, *t*(24) = 2.83, *p* < 0.01, 95% CI [0.02, 0.12], and the recognition of a sad expression (*M* = 577, *SD* = 194) was faster than that of a happy expression (*M* = 630, *SD* = 191) on the unattractive faces, *t*(24) = 2.10, *p* < 0.05, 95% CI [0.00, 0.08]. Under the familiar face condition, there was no significant interaction between attractiveness and expression [*F*(1, 24) = 1.35, *p* = 0.26] (see Figure 4).

#### Accuracy

An analysis of the error rate showed that the main effect of familiarity was not significant, *F*(1, 24) = 0.07, *p* = 0.79; the main effect of attractiveness was not significant, *F*(1, 24) < 0.01, *p* = 0.99; and the main effect of expression was significant, *F*(1, 24) = 9.40, *p* < 0.05, *η*^2^ = 0.28. The interaction between familiarity and attractiveness was significant, *F*(1, 24) = 22.46, *p* < 0.001, *η*^2^ = 0.48. The follow-up paired sample *t*-test results showed that when presented with familiar attractive faces, expression recognition had a lower error rate, *t*(24) = 3.06, *p* < 0.01. The interaction between attractiveness and expression was significant, *F*(1, 24) = 84.97, *p* < 0.001, *η*^2^ = 0.78. The follow-up paired sample *t*-test found that happy expression recognition on the attractive faces had a lower error rate, *t*(24) = 8.85, *p* < 0.001; on the unattractive faces, sad expression recognition had a lower error rate, *t*(24) = 2.99, *p* < 0.01. The three factors of familiarity, attractiveness, and expression were not significant, *F*(1, 24) = 0.04, *p* = 0.85.

In Experiment 2a, we found that facial attractiveness had different effects on expression recognition under different levels of facial familiarity. Under the familiar face condition, the influence of attractiveness on expression recognition was weakened or even unaffected. This result suggests that familiarity can modulate the effects of facial attractiveness on expression recognition in a static context, which is consistent with our expectations.

## Experiment 2b

The purpose of Experiment 2b was to explore whether the effects of familiarity and attractiveness on expression recognition found in Experiment 2a appeared in a dynamic context. Similarly, we expected that under the familiar face condition, the influence of attractiveness on expression recognition should be weakened or even unaffected. Thus, under the familiar face condition, the interaction between attractiveness and the expression recognition should not be significant.

### Method

#### Participants

In total, 29 new Chinese university students (18 females, *M* = 20.90 years, *SD* = 2.47 years) completed a morph movies task consistent with Experiment 1b. Similarly, based on a *post hoc* power analysis (*α* of 0.05, *η*^2^ = 0.50, G*Power 3.1), we found that this sample size yielded a high power of 1 − *β* = 0.84. All participants were right-handed and had normal or corrected-to-normal vision.

#### Materials and Procedure

##### Materials

We used the same 40 face models used in Experiment 2a. Then, we created 80 film clips by using the method described in Experimental 1b.

##### Procedure

Experiment 2b closely followed the procedure used in Experiment 1b, except for the familiar face stimuli used.

### Results and Discussion

Before the analysis, data from one participant with over 25% missing data were excluded (33.75% of the trials). Therefore, the final analysis included data from 28 participants. In addition, errors (incorrect button presses; 5.36% of the trials) and invalid responses (3.57% of the trials) were excluded from the response time analysis.

The dependent variable in this study was the average reaction time to identify the onset of the second expression in the film clips. The mean log-transformed response latencies were subjected to a 2 (attractiveness: attractive vs. unattractive) × 2 (expression: happy vs. sad) × 2 (familiarity: familiar vs. unfamiliar) mixed-model ANOVA. The spherical test *p* < 0.05 in the statistical test results indicated that the data did not satisfy the spherical hypothesis; therefore, the Greenhouse-Geisser corrected result is reported. The results showed a significant main effect of familiarity, *F*(1, 27) = 6.85, *p* < 0.05, *η*^2^ = 0.20; a significant main effect of attractiveness, *F*(1, 27) = 4.69, *p* < 0.05, *η*^2^ = 0.15; and no significant main effect of expression, *F*(1, 27) = 3.58, *p* = 0.07, *η*^2^ = 0.12.

**Figure 5:**
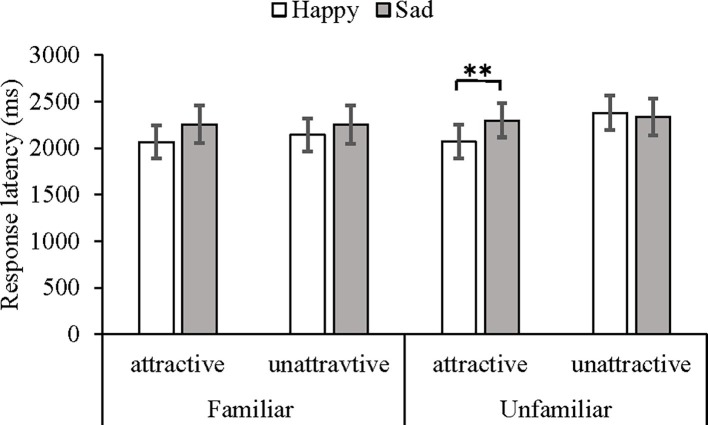
Mean response latency and SEs in milliseconds under different conditions of familiarity, attractiveness, and expression as measured by the morph movies task. **p < 0.01.

Importantly, there was a significant three-way interaction among attractiveness, expression, and familiarity, *F*(1, 24) = 4.28, *p* < 0.05, *η*^2^ = 0.14. Under the unfamiliar face condition, the *post hoc* tests, i.e., paired *t*-tests, showed that the recognition of happy expressions (*M* = 2,069, *SD* = 958) was faster than that of sad expressions (*M* = 2,296, *SD* = 962) on the attractive faces, *t*(27) = 4.05, *p* < 0.01, 95% CI [0.03, 0.09]. The difference between the happy (*M* = 2,379, *SD* = 987) and sad (*M* = 2,334, *SD* = 1,045) expression recognition on the unattractive faces was not significant, *t*(27) = 1.30, *p* = 0.21, 95% CI [−0.01, 0.05]. Under the familiar face condition, there was no significant interaction between attractiveness and expression [*F*(1, 27) =1.33, *p* = 0.26] (see Figure 5).

#### Accuracy

An analysis of the error rate showed that the main effect of attractiveness was significant, *F*(1, 27) = 7.90, *p* < 0.01, *η*^2^ = 0.23; the main effect of familiarity was not significant, *F*(1, 27) = 3.12, *p* = 0.09; and the main effect of expression was not significant, *F*(1, 27) = 0.34, *p* = 0.56. The three factors of familiarity, attractiveness, and expression were significant, *F*(1, 27) = 26.82, *p* < 0.001, *η*^2^ = 0.50. The follow-up paired sample *t*-test results showed that when presented with familiar attractive faces, the error rate of happy expression recognition was lower, *t*(27) = 3.22, *p* < 0.01. Under the familiar unattractive face conditions, there was no difference in the error rate between happy and sad expression recognition, *t*(27) = 0.67, *p* = 0.51. Happy expression recognition on the unfamiliar attractive faces exhibited a lower error rate, *t*(27) = 5.57, *p* < 0.001, and sad expression recognition on the unfamiliar unattractive faces exhibited a lower error rate, *t*(27) = 8.46, *p* < 0.001.

## General Discussion

Following the debate between classic and dynamic theories of face perception, the purpose of the current research was to investigate whether facial attractiveness influenced expression identification. In addition, we explored the role of familiarity in this effect.

The first goal of the present research was to investigate whether facial attractiveness affects expression recognition in both static and dynamic contexts. The results show a significant interaction between facial attractiveness and expression recognition and suggest that facial attractiveness influenced the participants’ identification of facial expressions, which was reflected in the fact that the participants were able to recognize the happy expressions on attractive faces more quickly, further demonstrating the advantage of happy expression recognition. The recognition of attractive faces and happy expressions may offer benefits *via* rewards, which could facilitate their rapid recognition (Chatterjee et al., 2009; Golle et al., 2014; Zhang et al., 2017, 2019). In the absence of attractive faces, this advantage may be lacking in both dynamic and static experimental contexts, which is consistent with the prediction of the dynamic theory of face perception. In addition, the predicted interaction between facial attractiveness and facial expression was found, which is consistent with the results reported in previous studies (Golle et al., 2014; Lindeberg et al., 2018) and the prediction of the dynamic theory of face perception.

The second goal of the present research was to investigate whether visual familiarity and facial attractiveness simultaneously moderate expression recognition. Experiments 2a and 2b found that familiarity altered the influence of the attractiveness of the target face on expression recognition. Specifically, we found that under the familiar face condition, attractiveness did not affect expression recognition. Thus, the influence of familiarity on expression recognition was greater. Under the effect of familiarity, the influence of attractiveness on expression recognition may be weakened or even unaffected. We speculate that this finding may be related to the effect of familiarity on facial expression recognition because familiarity increases the fluency of facial expression processing and makes it easier to process expressions on familiar faces such that they can be recognized by the perceiver more quickly (Jacoby and Dallas, 1981; Bornstein and D’Agostino, 1994; Claypool et al., 2007). One possible reason might be that familiarity affects the subjective feelings of facial attractiveness. According to the mere exposure effect proposed by Zajonc (1968), when an unattractive face has been seen for a long period of time, it is subjectively perceived in a manner that increases its level of attractiveness. Therefore, when the visual familiarity of the face is enhanced, the difference in the level of attractiveness of the face is not particularly obvious. Notably, this possible explanation requires additional investigation in future research. Future research is needed to further examine the interaction between facial attractiveness and familiarity in expression recognition and verify this possibility.

In general, this research examines whether attractiveness affects expression recognition. Previous studies have used static situations (Taylor and Bryant, 2016; Lindeberg et al., 2018). In our research, both static and dynamic contexts were used to increase ecological validity. Furthermore, the results show that the relationship between attractiveness and expression recognition is also consistent with the theory of dynamic face perception and provides more evidence supporting this theory. This research also expands the category of attractiveness that affects expressions and makes certain contributions to research in the field of face perception. In addition, this research examines whether familiarity and attractiveness affect expression recognition. It is found that under the familiar face condition, the influence of attractiveness on expression recognition is not very strong. In this respect, this research has a certain degree of innovation.

However, this research has the following limitations. First, this research only examined the expressions of happiness and sadness. Facial expressions also include many other types, such as surprise, disappointment, and fear. Future research could consider exploring the relationship between facial attractiveness and expression recognition and the rich relationship between facial attractiveness and expression recognition. Second, in this research, static and dynamic contexts were used as different experimental conditions to examine the relationship between attractiveness and expression recognition. Each study involved different participants. Future research could also consider static and dynamic contexts as internal factors to investigate the relationships among attractiveness, familiarity, and expression recognition. Finally, although this research provides an initial examination of the impact of familiarity and attractiveness on expression recognition, the specific mechanisms need to be further explored. For example, future research could deeply explore the cognitive neural mechanism of familiarity and attractiveness affecting expression recognition from the perspective of cognitive neuroscience.

In conclusion, this research demonstrates that the ability to categorize other people’s facial expressions is influenced by the attractiveness of the face in both static and dynamic experimental contexts. The interaction between facial attractiveness and expression identification suggests that facial attractiveness may affect expression identification, which is consistent with the dynamic theory of face perception. More specifically, we find that happy expressions on attractive faces can be recognized more quickly, highlighting the advantage of happy expression recognition in both static and dynamic contexts. However, when introducing familiar faces, the advantage of such happy expression recognition was weakened. Thus, in static and dynamic familiar face contexts, attractiveness does not strongly affect expression recognition, and the influence of familiarity is greater. This finding also reflects the fact that under the influence of familiarity, the influence of attractiveness on expression recognition may be weakened or even unaffected. Our research is the first to examine the relationship between facial attractiveness and expression recognition in a dynamic context. In addition, we find that familiarity can modulate the effects of facial attractiveness on the identification of facial expression in both static and dynamic contexts, emphasizing the importance of familiarity in visual cognition.

## Data Availability Statement

All datasets generated for this study are included in the article/supplementary material.

## Ethics Statement

The Institutional Review Board of South China Normal University that approved the study. Consent procedure used for human participants. This study was carried out in accordance with the recommendations of “Human Research Ethics Committee for Non-Clinical Faculties, Institutional Review Board of South China Normal University” with written informed consent from all subjects. All subjects gave written informed consent in accordance with the Declaration of Helsinki. The protocol was approved by the “Institutional Review Board of South China Normal University”.

## Author Contributions

XH and LZ conceived and designed the research. LZ participated in the data collection. JL participated in the data analysis and data interpretation. JL and DH wrote the paper. JL and XH participated in the revision of the article. XH, XZ, TZ, and WZ helped provide constructive advice. XH supervised the entire project.

### Conflict of Interest

The authors declare that the research was conducted in the absence of any commercial or financial relationships that could be construed as a potential conflict of interest.

## Footnotes

^1^http://www.baidu.com/

^2^www.facegen.com

^3^http://www.fantamorph.com/index.html
